# From Genome to Function: Systematic Analysis of the Soil Bacterium *Bacillus Subtilis*

**DOI:** 10.1002/cfg.69

**Published:** 2001-02

**Authors:** Colin R. Harwood, Samuel G. Crawshaw, Anil Wipat

**Affiliations:** 1 Department of Microbiology and Immunology University of Newcastle upon Tyne Framlington Place Newcastle upon Tyne NE2 4HH UK; 2 Department of Agricultural and Environmental Sciences University of Newcastle upon Tyne King George VI Building Newcastle upon Tyne NE1 7RU UK

## Abstract

*Bacillus subtilis* is a sporulating Gram-positive bacterium that lives primarily in the soil and associated water sources. Whilst this bacterium has been studied extensively in the laboratory, relatively few studies have been undertaken to study its activity in natural environments. The publication of the *B. subtilis* genome sequence and subsequent systematic functional analysis programme have provided an opportunity to develop tools for analysing the role and expression of *Bacillus* genes *in situ*. In this paper we discuss analytical approaches that are being developed to relate genes to function in environments such as the rhizosphere.


**Comparative and Functional Genomics**
*Comp Funct Genom* 2001; **2**: 22–24.

Conference Paper

### From genome to function: systematic analysis of the soil bacterium *Bacillus subtilis*

Colin R. Harwood[1]*, Samuel G. Crawshaw[1] and Anil Wipat[2]
[1] *Department of Microbiology and Immunology, University of Newcastle upon Tyne, Framlington Place, Newcastle upon Tyne NE2 4HH, UK*
[2] *Department of Agricultural and Environmental Sciences, University of Newcastle upon Tyne, King George VI Building, Newcastle upon Tyne NE1 7RU, UK*

**Correspondence to:
C. R. Harwood, Department of Microbiology and Immunology, University of Newcastle upon Tyne, Framlington Place, Newcastle upon Tyne NE2 4HH, UK.*

### Abstract

***Bacillus subtilis* is a sporulating Gram-positive bacterium that lives primarily in the soil and associated water sources. Whilst this bacterium has been studied extensively in the laboratory, relatively few studies have been undertaken to study its activity in natural environments. The publication of the *B. subtilis* genome sequence and subsequent systematic functional analysis programme have provided an opportunity to develop tools for analysing the role and expression of *Bacillus* genes *in situ*. In this paper we discuss analytical approaches that are being developed to relate genes to function in environments such as the rhizosphere. Copyright © 2001 John Wiley & Sons, Ltd.**

**Keywords: DNA arrays; environmental marker cassette; functional analysis; gene expression; reporter genes; rhizosphere; signature-tagged mutagenesis**

*Bacillus subtilis* has been studied extensively over the past 50 years and is consequently regarded as a well established model for Gram-positive bacteria. The detailed knowledge of its biochemistry, genetics and physiology has arisen because of the unusually high genetic amenability of *B. subtilis* strain 168. *B. subtilis* and other *Bacillus* species are used in a wide range of industrial processes for the production of extracellular enzymes, vitamins and fine biochemicals (Harwood, 1992), and this industrial use has also enhanced our knowledge of the molecular and physiological characteristics of this bacterium.

*Bacillus subtilis* was the first Gram-positive, soil microorganism to have its genome completely sequenced (Kunst *et al.*, 1997). After the completion of the genome sequence, the resulting research momentum was harnessed to establish a number of systematic research programmes in Europe and Japan. These collaborative programmes, involving industry and academia, were designed to expand knowledge of the molecular biology of strain 168, ultimately to produce a highly detailed mechanistic model of its behaviour in laboratory-based studies. The EU-funded consortia are combined under the umbrella of BACELL (**http://www.ncl.ac.uk/bacell/**) and include: (a) a functional analysis programme that has led to the construction and phenotypic characterization of a set of isogenic mutants for each gene of unknown function; (b) a programme aimed at defining the *B. subtilis* secretome and adapting it for the high-level production of heterologous proteins (**http://www.ncl.ac.uk/ebsg/**); (c) a genome-minimizing programme designed to maximize the fermentation efficiency of *B. subtilis*; and (d) a programme designed to model regulatory networks in *B. subtilis* through analysis of global gene expression under various growth conditions (**http://www.ncl.ac.uk/bacellnet/**). Data resulting from these consortia have been compiled into databases, access to which is freely available over the Internet. These include: Subtilist (**http://genolist.pasteur.fr/SubtiList/**), a dedicated DNA sequence database; Micado (**http://locus.jouy.inra.fr/cgi-bin/genmic/madbase/progs/madbase.operl/**), which has data on the characterization of the isogenic mutant collection; and Sub2D (**http://microbio2.biologie.uni-greifswald.de:8880/**), which holds data on the analysis of the *B. subtilis* 168 proteome. A new database, Subscript, is planned to store and analyse data on the transcriptome.

Despite its widespread occurrence in nature, very

Copyright © 2001 John Wiley & Sons, Ltd.

few studies have been undertaken to examine the behaviour of this *B. subtilis* in its natural habitat, the soil, or to consider how its genetic characteristics have been moulded by the demanding nature of this environment. However, commercial interest in the agricultural applications of this bacterium is currently on the increase (**http://www.attra.org/attra-pub/ipm.html**) and strains of *B. subtilis* are already used extensively as biological control agents and plant growth-promoting rhizobacteria (Brannen and Kenney, 1997). Studies directed at further understanding the molecular ecology of *Bacillus* by analysing gene function in the soil are now needed to enhance the agricultural applications of this species. The *Bacillus* community is now well-placed to perform such studies by capitalizing on the extensive knowledge-base and resources that have accumulated on the biology of this bacterium.

Molecular techniques for studying the ecology of Gram-negative bacteria, such as the pseudomonads, are well advanced in comparison to those of Gram-positive bacteria. However, much of this technology is applicable to studying the environmental genomics of *Bacillus* species. In addition, new technologies for performing molecular analyses at a genomic level are emerging with the exploitation of the *B. subtilis* genome sequence data. Technologies for post-genomic analysis, such as the use of DNA arrays (Duggan *et al.*, 1999) for characterization of the transcriptome, will ultimately facilitate more detailed studies on genes required for survival and fitness in natural environments. Strains of *B. subtilis* appear to be adapted to specific environmental niches (e.g. endophytes), and the use of DNA arrays for comparative genomics is likely to facilitate the identification of the relevant distinguishing genetic features.

One existing approach to studying gene function in natural environments is to identify genes that are specifically expressed in natural habitats. Techniques for identifying promoters that are expressed under natural environments, such as IVET (Rainey *et al.*, 1997), have been shown to identify genes that allow bacteria to colonize particular environments (Rainey *et al.*, 2000). Although an IVET system has not yet been developed for use in *Bacillus* species, promoter trapping techniques that allow the identification of genes which are transiently or conditionally repressed *in vitro* have been developed for *B. subtilis* (Salamitou *et al.*, 1997). Such techniques may prove to be directly applicable for use in natural systems with little modification.

Insertional mutagenesis has also proved to be a powerful approach to the determination of gene function in bacteria. To date most of these studies have been performed *in vitro*, although the value of this technique for identifying genes required for the colonization of the rhizosphere has recently been demonstrated for *Pseudomonas fluorescens*. Dekkers and co-workers (Dekkers *et al.*, 1998a, b, c, 2000) have screened banks of random mutants created by transposon mutagenesis in a gnotobiotic model system (Simons *et al.*, 1996) and a number of genes that are important for rhizosphere colonization were identified. The existence of a set of defined, isogenic mutants in all genes of unknown function of *B. subtilis* promises to be a useful resource for studying phenotypic alterations that affect the ability of this organism to colonize its natural environment. Significantly, the use of targeted mutants obviates the need for the lengthy procedures required to identify genes inactivated in randomly-generated mutants.

Work in our laboratory has been directed at developing suitable tools for the functional analysis of *B. subtilis* genes in natural environments. We have constructed a series of multi-functional cassettes that allow gene function to be investigated *in situ* by insertional mutagenesis and the analysis of gene expression. These cassettes enhanced the utilization of the existing bank of *B. subtilis* isogenic mutants for *in situ* investigations by the replacement of the *lacZ* reporter gene in pMutin (Vagner *et al.*, 1998) by homologous recombination. In addition, vectors incorporating the cassette have been developed to facilitate the targeted mutagenesis of specific genes. The cassette includes a *gfp* reporter gene to allow gene expression to be monitored by fluorescence microscopy or spectrometry. Three additional features are designed to aid the isolation and quantification of specific strains in a soil environment: a unique signature tag, a chloramphenicol resistance marker, and a catechol 2,3-dioxygenase gene (*xylE*), enabling identification and enumeration of mutants both on selective agar plates and *in situ* by signature-tagged mutagenesis (Hensel *et al.*, 1995). The unique signature tag also facilitates the measurement of gene expression *in situ* by quantitative RT–PCR.

One-third of the genes on the *B. subtilis* genome are of unknown function and it is likely that a

Copyright © 2001 John Wiley & Sons, Ltd.

significant number of these are required for stress resistance and survival in its natural environment. Indeed, data emerging from the systematic programmes outlined above suggest that a significant proportion of the *B. subtilis* genome is dedicated to growth and survival in the extremely variable conditions found in the soil and rhizosphere. Thus, knowledge of the behaviour of *B. subtilis* in its natural environment is likely to be of increasing importance for elucidating the role of genes currently of unknown function.

### References

Brannen PM, Kenney DS. 1997. Kodiak—a successful biological-control product for suppression of soil-borne plant pathogens of cotton. *J Indust Microbiol Biotechnol* **19**: 169–171.

Dekkers LC, Bloemendaal CJP, de Weger LA, *et al*. 1998a. A two-component system plays an important role in the root-colonizing ability of *Pseudomonas fluorescens* strain WCS356. *Mol Plant Microb Interact* **11**: 45–56.

Dekkers LC, Phoelich CC, van der Fits L, Lugtenberg BJJ. 1998b. A site-specific recombinase is required for competitive root colonization *by Pseudomonas fluorescens* WCS365. *Proc Natl Acad Sci U S A* **95**: 7051–7056.

Dekkers LC, van der Bij AJ, Mulders IH, *et al*. 1998c. Role of the O-antigen of lipopolysaccharide, and possible roles of growth rate and of NADH : ubiquinone oxidoreductase (nuo) in competitive tomato root-tip colonization by *Pseudomonas fluorescens* WCS365. *Mol Plant Microb Interact* **11**: 763–771.

Dekkers LC, Mulders IHM, Phoelich CC, *et al*. 2000. The *sss* colonization gene of the tomato–*Fusarium oxysporum* f. sp *radicislycopersici* biocontrol strain *Pseudomonas fluorescens* WCS365 can improve root colonization of other wild-type *Pseudomonas* spp. bacteria. *Mol Plant Microb Interact* **13**: 1177–1183.

Duggan DJ, Bittner M, Chen Y, Meltzer P, Trent JM. 1999. Expression profiling using cDNA microarrays. *Nature Genet* **21**: 10–14.

Harwood CR. 1992. *Bacillus subtilis* and its relatives: molecular biological and industrial workhorses. *Trends Biotechnol* **10**: 247–256.

Hensel M, Shea JE, Gleeson C, Jones MD, Dalton E, Holden DW. 1995. Simultaneous identification of bacterial virulence genes by negative selection. *Science* **269**: 400–403.

Kunst F, Ogasawara N, Moszer I, *et al*. 1997. The complete genome sequence of the Gram-positive bacterium *Bacillus subtilis*. *Nature* **390**: 249–256.

Rainey PB, Heithoff DM, Mahan MJ. 1997. Single-step conjugative cloning of bacterial gene fusions involved in microbe–host interactions. *Mol Gen Genet* **256**: 84–87.

Rainey PB, Preston GM. 2000. *In vivo* expression technology strategies: valuable tools for biotechnology. *Curr Opin Biotechnol* **11**: 440–444.

Salamitou S, Agaisse H, Lereclus D. 1997. A genetic system that reports transient activation of genes in *Bacillus*. *Gene* **202**: 121–126.

Simons M, van der Bij AJ, Brand I, *et al*. 1996. Gnotobiotic system for studying rhizosphere colonization by plant growth-promoting *Pseudomonas* bacteria. *Mol Plant Microb Interact* **9**: 600–607.

Vagner V, Dervyn E, Ehrlich SD. 1998. A vector for systematic gene inactivation in *Bacillus subtilis*. *Microbiology* **144**: 3097–3104.

Copyright © 2001 John Wiley & Sons, Ltd. 
